# Supplementary material for: A machine learning approach to integrate big data for precision medicine in acute myeloid leukemia
Source: Nat Commun. 2018 Jan 3;9:42. doi: 10.1038/s41467-017-02465-5 (PMC5752671; doi:10.1038/s41467-017-02465-5)
Supplement: Supplementary file 1 — Supplementary Information [file 41467_2017_2465_MOESM1_ESM.pdf]

Supplementary Information

Supplementary Notes

Supplementary Note 1: mRNA expression profiles

For all of the 30 samples, gene expression was analyzed using Affymetrix Human U133 Plus 2.0 arrays. The analysis was performed at the Department of Environmental and Occupational Health Science's Functional Genomics Core Laboratory at the University of Washington. RNA was isolated using the Qiagen RNeasy Mini kit (Qiagen Inc, Germantown MD). RNA quantity was assessed by measuring OD_{260}, and purity by $OD_{260/280}$ and $OD_{260/230}$ ratios with the NanoDrop spectrophotometer (Thermo Fisher Scientific Inc, Wilmington, DE). RNA integrity was assessed using the Agilent 2100 Bioanalyzer (Agilent Technologies Inc., Santa Clara, CA). RNA samples were required to meet these stringent quality control parameters and were then processed according to the manufacturer's recommendations. Briefly, 250 ng of total RNA was reverse transcribed. The resulting cDNA was converted to biotinylated cRNA. The biotinylated fragments were hybridized to Affymetrix U133 Plus 2.0 arrays, washed and stained. The arrays were scanned with an Affymetrix GenChip® 3000 scanner. Image generation and feature extraction were performed using Affymetrix GeneChip Command Console software.

For the additional test set of 12 patient samples (from which we show the results in **Fig. 3b**), RNA-seq data generation was performed at the Northwest Clinical Genomics Laboratory, UW Medicine Center for Precision Diagnostics using the TruSeq® stranded mRNA kit from Illumina (San Diego, CA) according to manufacturer's instructions. We had two RNA-seq replicates for each of the 12 samples. For MERGE experiments, we averaged the FPKM (Fragments Per Kilobase of transcript per Million mapped reads) values from the Cufflinks[1] output from the two replicates of each sample.

Gene expression for the 14 AML cell lines (EOL-1, MOLM/16, NB4, OCI-AML3, SKM1, HL60, KG1, MOLM/13, MV4.11, OCI-AML2, PL-21, U937, KASUMI1, THP-1) was retrieved from the Cancer Cell Line Encyclopedia (CCLE)[2].

Supplementary Note 2: Curve fitting to estimate drug sensitivity profiles

As was done in CCLE study[2], from each does-response data for a combination of a drug and a patient sample, we extracted summary statistics, including area under the curve (AUC), half maximal inhibitory concentration (IC_{50}), half maximal effective concentration (EC_{50}), and maximal effect level (A_{max}). In brief, we fitted the dose-response data (averaged over duplicates) to the following 4 parameter sigmoid model:

$$y = A_{\mathrm{inf}} + \left(\frac{A_0 - A_{\mathrm{inf}}}{1 + \left(\frac{x}{\mathrm{EC}_{50}}\right)^{\mathrm{Hill}}} \right)$$

Here, A_0 and A_{inf} are the top and bottom asymptotes of the response (cell viability); EC_{50} is the inflection point of the curve; and Hill is the Hill slope, which describes the curve's steepness. Other key parameters derived from the models include IC_{50} (the concentration where the fitted curve crosses 50% in cell viability) and A_{max} (the maximal activity value reached within a model). We extracted these parameters (AUC, IC_{50}, EC_{50}, and A_{max}) after curve fitting by using MATLAB's 'nlinfit' function for nonlinear curve fitting. For inactive compounds, it is impossible to derive an IC_{50}; in this instance, as was done in the CCLE study[2], we simply used the maximum tested concentration as the default value – which serves primarily as a placeholder to allow algorithms to work on all samples. Batch effects were corrected using ComBat[3] for each drug sensitivity summary based on the three batches of experiments for the 30 patients.

From these statistics, we chose AUC because it represents an average of drug sensitivity across a range of drug concentrations. Indeed, AUC showed by far the strongest association with gene expression levels; the number of significant associations (FDR corrected p-value < 0.1) between a gene and a drug sensitivity measure was 53,967 for AUC, 23,641 for IC_{50}, 15,112 for EC_{50}, and 7,132 for A_{max}. Considering additional drug sensitivity measures would increase the total number of hypotheses.

Supplementary Note 3: Summary of the clinical information and its consistency with our *in vitro* drug sensitivity data

Of the 30 AML patient samples, 24 were newly diagnosed, and 6 had relapsed. The majority of samples (19 of 30) were males. The median age was 54, and the age range was [19, 83]. Six patients had antecedent hematologic disorders. According to European LeukemiaNet criteria[4], seven samples were in favorable risk group, eleven were in intermediate-1, 2 were in intermediate-2, and ten were in the adverse risk cytogenetics group. Several different regimens were used to treat the patients, most of which included cytarabine, and many of which included an anthracycline. **Supplementary Data 3** contains the detailed clinical information including usual evaluation (including risk group category and cytogenetic features), response to treatment, duration of remission, and the individual regimens for each of the samples.

We measured the standard clinical mutation status on *FLT3* and *NPM1* for most of the 30 patients (26 patients tested for *NPM1* mutation; 27 tested for *FLT3* mutation). Seven of 26 patients had an *NPM1* mutation, and seven of 27 patients had the *FLT3* ITD mutation. We observed a statistically significant association between *FLT3* mutation status and 12 drugs (FDR corrected p-value < 0.1): AS101, AT-7519, AZD7762, cladribine, mitomycin C, mitoxantrone, NVP-AUY-922, obatoclax, PIK-75, midostaurin (PKC412), sunitinib, and tandutinib. Three of these drugs (midostaurin, sunitinib and tandutinib) are known to have a *FLT3* inhibitory role. None of the drugs is associated with *NPM1* mutation status at the same significance level.

Additionally, we checked the statistical significance of the association between the complete remission (CR) status and the drug sensitivity measure (AUC) across all 53 drugs. Interestingly, 12 of 15 drugs (80%) (azacitidine, bortezomib, cladribine, clofarabine, daunorubicin, etoposide, fludarabine, mitoxantrone, panobinostat, PKC412, tretinoin, vorinostat) that have shown clinical efficacy in AML treatment were significantly associated with CR at FDR= 0.05, while only 21 of the other 38 drugs (55.3%) showed significant association with CR (**Table S2**). Some of the 38 drugs in the latter set have successor agents that have shown clinical efficacy in AML, such as the Bcl2 inhibitor ABT-199 (venetoclax), a successor to ABT-737 and ABT-263 that we tested. Moreover, drugs might have additive or synergistic activity in combinations; however, in this study, the drugs were assayed as single agents. We note that cytarabine, commonly used to treat AML, is not listed in **Table S2** because it was not selected by the procedure that we followed to select the 53 drugs we focused on in our computational framework.

While cytarabine is commonly used to treat AML and a majority of the 30 AML patients received this drug, it was not one of the 53 drugs included in our MERGE computational framework: our criterion for selecting whether to include each of the 160 tested drugs in the study was exhibited activity (cell viability ≤ 50%) of the drug against at least half of the 30 patient samples; cytarabine did not satisfy this criterion. One possible reason is that the tested concentrations were too low for the 12 patients in the first batch (concentration range 2×10^{-10} M to 1×10^{-6} M), and cytarabine did not exhibit any activity for those patients until the highest concentration point. The concentrations were then increased for the next 18 patient samples (range 4×10^{-8} M to 1×10^{-4} M). For comparison, the peak mean plasma concentration after high dose cytarabine was 2×10^{-3} M[5].

Supplementary Note 4: Extracting driver features from publicly available sources

The MERGE algorithm takes a set of *driver features* for each gene – namely, expression hubness, candidate regulators, mutation, copy number variation, and methylation – as input and estimates a MERGE score for each gene based on these features (**Fig. 1b**). These features were extracted from publicly available sources, such as TCGA (The Cancer Genome Atlas) AML study[6], AML expression studies[7], and gene annotation databases, as described in detail below.

Expression hubness: In our prior study, we developed a novel computational method, named SPARROW (**SPAR**se selected exp**R**essi**O**n regulators identified **W**ith penalized regression), to estimate each gene's *hubness* purely based on expression data from cancer patients. SPARROW employs a sparse statistical model in which each gene's expression level is modeled as a linear combination of a small set of other genes (i.e., sparse basis), and determines the hubness of each gene based on how often it is chosen in the sparse basis for any other gene[8]. To use gene hubness as a MERGE feature, we downloaded the R data object (.rda) containing the SPARROW results for AML from http://sparrow-leelab.cs.washington.edu/data. We then used 'sparrow1' scores (the number of downstream genes) from the 'basesFreq' object as the expression hubness feature.

Candidate regulators: The genes known to regulate other genes are more likely disease drivers and hence more reliable molecular markers for therapeutic response than those that are not. To incorporate this

hypothesis, we used a list of genes known to have regulatory roles, including transcription factors, chromatin remodelers and signal transduction genes, constructed based on gene annotation databases[7]. Based on this list of 3,052 genes, we generated a binary feature for each gene by assigning 1 if the gene was on the list and 0 otherwise.

Mutation: We downloaded significance measures of mutation frequencies for each gene measured in the MutSig2CV Analysis of the AML study from TCGA (http://firebrowse.org/?cohort=LAML). Each gene was given a p-value that measured the statistical significance that the gene had mutated more often than expected by chance given background mutation processes across patients; we used $-\log_{10}$p-value as the feature value.

Copy number variation (CNV): We downloaded CNV measures (gdac.broadinstitute.org_LAML-TB.CopyNumber_Gistic2.Level_4.2015082100.0.0.tar.gz) from http://gdac.broadinstitute.org/runs/analyses__2015_08_21/data/LAML/20150821/. Then, we used all_data_by_genes.txt in this tar.gz file to assign 1 (having CNV) or 0 (no CNV) to each gene. We set the CNV feature of a gene to 1 if the gene was amplified or deleted by at least .05 in at least 20 of 191 patients (~10%), and to 0 otherwise.

Methylation: We downloaded DNA methylation measures (gdac.broadinstitute.org_LAML.Methylation_Preprocess.Level_3.2015110100.0.0.tar.gz) from http://gdac.broadinstitute.org/runs/stddata__2015_11_01/data/LAML/20151101/. We then used LAML.meth.by_mean.data.txt in this tar.gz file to obtain the average methylation levels for each gene across all patients.

Supplementary Note 5: Averaging the methylation values across the CpG probes and across the samples

The AML study from TCGA includes preprocessed methylation profiles generated by four different pipelines that retrieve the gene-based methylation values from the CpG probes. These four include: (1) using the CpG probe with the highest anticorrelation with the gene's expression level, (2) using the CpG probe with the highest anticorrelation with the clinical data, (3) using the mean signal intensities across all CpG probes in proximity to the gene, and (4) using the CpG probe with the maximum standard deviation across all beta values.

A specific position of methylation (e.g., whether it occurs around the transcription start site or over a gene body) offers important information to understand the epigenetic cause of variation of downstream phenotypes, such as gene expression levels. Accordingly, the data generated by the first two preceding approaches were generally chosen when the goal was to explain a phenotype of interest (i.e., gene expression or clinical data) and to choose the probe likely to represent the molecular basis for the phenotype. Since we sought to develop a general framework to utilize methylation data from the external AML sources, our study used the data generated by the approach (3), which is independent from any specific phenotype and provides a general summary of TCGA methylation data. We did not use the data from approach (4) because it discards probes with a standard deviation below a specified cutoff, so the dataset contained only ~2K genes, too small compared to the ~17K genes in the MERGE model.

After getting each sample's (patient's) methylation values for each gene as explained above, we used the sample mean across patients to compute each gene's methylation score. While other ways may provide a summary statistic across all patients, we considered only the sample mean in the current study: mean is the most commonly used measure of central tendency for continuous-valued variables, and several authors have averaged methylation values over samples (e.g., Moarii, Boeva, Vert, & Reyal, 2015).

Supplementary Note 6: Standardization of driver features, predictors and response variables

We standardized each driver feature before running the MERGE algorithm so that each feature had mean 0 and variance 1. We performed standardization so that despite different scales, driver features were treated fairly when estimating MERGE scores. We standardized binary features (candidate regulator and CNV) as we did other non-binary features. For any statistical model that combines data from several different kinds of variables (i.e., features) and aims to learn weights for the variables with regularization, one must provide all variables to the algorithm on the same scale to achieve fairness[10]. Binary variables are not exceptions[11].

We also standardized predictor variables (gene expression levels) and response variables (drug sensitivity measures) for MERGE and all other methods compared to MERGE in our experiments: ElasticNet, multi-task learning, Pearson's p-value, Spearman p-value, and Bayesian multi-task multiple kernel learning (MKL).

Supplementary Note 7: Measuring the significance of association between a gene expression level and a drug sensitivity measure

We computed the p-value of association between each gene and each drug using t statistics and associated p-values, measuring the correlation between the gene and drug in a univariate linear regression model. Then, we applied FDR correction for multiple hypotheses for all pairs of genes and drugs, using the Benjamini & Hochberg (1995) method. We considered the (genome-wide) FDR corrected p-value < 0.1 to be significant throughout the paper. When we measured the consistency of gene-drug associations in CCLE data, we used an uncorrected p-value of 0.1 as a cutoff to indicate that a gene-drug association discovered in patient data was replicated in validation data. We did not apply the FDR correction method in validation data because the FDR correction process is designed to maintain a certain FDR level when making a large number of hypotheses, and we do not make hypotheses during validation.

Supplementary Note 8: The MERGE algorithm

To learn the MERGE scores, we developed a probabilistic graphical model approach that provides a statistical model to represent relationships among variables in input data and a principled way of learning the parameters of the model from data[13]. Let w_{ij} represent the magnitude and sign of the impact of gene i on drug j. This relationship can be modeled as: $\mathrm{y}_j \sim \mathcal{N}(w_{ij}x_i, \mathcal{E}^2)$, where x_i represents the expression level of gene i and y_j represents the drug response measure (i.e., AUC) of drug j. A conventional statistical model widely used in a genome-wide association analysis does not model a

marker potential (**Fig. 1a**). In the conventional model, we estimate w_{ij} for each combination of a gene and a drug by finding w_{ij}, which minimizes the squared error of prediction across all samples (here, patients):

$$\underset{w_{ij}}{\text{minimize}} \sum_{n\in\{\text{all patients}\}} (y_j^{(n)} - w_{ij} x_i^{(n)})^2, \qquad \text{Eq (1)}$$

where (n) means the nth sample. Then, the learned w_{ij} value would equal Pearson's correlation coefficient between the two variables, x_i and y_j, when both x_i and y_j were scaled to have the same standard deviance (specifically through the common standardization procedure).

MERGE's key innovation is the modeling of the *prior probability distribution* over each w_{ij} such that it incorporates prior knowledge on gene i in terms of its potential to be a molecular marker. We model the prior distribution over each w_{ij} as a normal distribution $P(w_{ij}) \sim \mathcal{N}(0, \sigma_i^2)$, where σ_i^2 represents a prior variance specific to gene i. If gene i has a low value on σ_i^2, the weight value w_{ij} would be inclined to be close to zero (i.e., low marker potential); if gene i has a high value on σ_i^2, then, since there is more probability mass away from zero, the weight value w_{ij} would be more inclined to deviate from zero (i.e., high marker potential). We model σ_i^2 as a function of a weighted combination of its driver features $\sigma_i^2 = \frac{1}{\lambda - \sum_{k=1}^{5} v_k d_{ik}}$, where d_{ik} is the value of the kth driver feature on gene i. Genes with a high value of $\left(\sum_{k=1}^{5} v_k d_{ik}\right)$ would have a high variance σ_i^2 (i.e., high marker potential); thus, we define the MERGE score of gene i as $\left(\sum_{k=1}^{5} v_k d_{ik}\right)$. λ is a regularization parameter: when it is high, each σ_i^2 would take a lower value, which means a lower variance on the weight (w_{ij}) values associated with each gene.

We learn the parameters – weight values w_{ij} for each gene i and drug j, and the driver feature weights v_k – that optimize the joint log-likelihood function $\log P(\mathbf{X}, \mathbf{Y}, \mathbf{W}, \mathbf{V})$, where $\mathbf{X}$ is the expression data matrix (p genes × n patients), $\mathbf{Y}$ is the drug response data matrix (q drugs × n patients), $\mathbf{W}$ is the gene-drug weight matrix (p genes × q drugs) that contains w_{ij} for each gene i and drug j, and $\mathbf{V}$ is a vector that contains 5 driver feature weights. The MERGE algorithm can be seen as a process of projecting high-dimensional gene-drug associations (p genes × q drugs) onto a lower-dimensional space by constraining weight values based on prior information on genes' potential to drive the disease.

The objective function $\log P(\mathbf{X}, \mathbf{Y}, \mathbf{W}, \mathbf{V})$ can be decomposed as

$$\log[P(\mathbf{Y}|\mathbf{X}, \mathbf{W}, \mathbf{V}) P(\mathbf{X}|\mathbf{W}, \mathbf{V}) P(\mathbf{W}|\mathbf{V}) P(\mathbf{V})]$$

$$= \log P(\mathbf{Y}|\mathbf{X}, \mathbf{W}) + P(\mathbf{X}) + \log P(\mathbf{W}|\mathbf{V}) + \log P(\mathbf{V})$$

$$= \log P(\mathbf{Y}|\mathbf{X}, \mathbf{W}) + \log P(\mathbf{W}|\mathbf{V}),$$

assuming a uniform prior distribution over $\mathbf{X}$ and $\mathbf{V}$ (i.e., $P(\mathbf{X})$ and $P(\mathbf{V})$ are constant). The conditional log-likelihoods are:

$$\log P(\mathbf{Y}|\mathbf{X}, \mathbf{W}) = \log \prod_{i,j,n} \mathcal{N}\left(w_{ij} x_i^{(n)}, \mathcal{E}^2\right) = -\sum_{i,j,n} \left\{\log \sqrt{2\mathcal{E}^2\pi} + \frac{(y_j^{(n)} - w_{ij} x_i^{(n)})^2}{2\mathcal{E}^2}\right\},$$

$$\log P(\mathbf{W}|\mathbf{V}) = \log \prod_{i,j} \mathcal{N}\left(0, \frac{1}{\lambda - \sum_{k=1}^{5} v_k d_{ik}}\right) = -\sum_{i,j} \left\{\log \sqrt{\frac{2\pi}{\lambda - \sum_{k=1}^{5} v_k d_{ik}}} + \frac{(\lambda - \sum_{k=1}^{5} v_k d_{ik}) w_{ij}{}^2}{2}\right\},$$

for each gene i, drug j, and a sample n. Maximizing $\log P(\mathbf{X}, \mathbf{Y}, \mathbf{W}, \mathbf{V})$ with respect to $\mathbf{W}$ and $\mathbf{V}$ leads to the following optimization problem when constants are dropped and $\mathcal{E}^2$ is set to 1 (since we

standardize **X** and **Y** before we apply the algorithm, $\mathcal{E}^2 = 1$ is a reasonable choice that simplifies the optimization).

$$\underset{w_{ij}\in\mathbf{W},\, v_k\in\mathbf{V}}{\text{minimize}} \sum_{i,j}\left\{\sum_n (y_j^{(n)} - w_{ij}x_i^{(n)})^2\right\} + \sum_{i,j}\left(\lambda - \sum_{k=1}^{5} v_k d_{ik}\right) {w_{ij}}^2 - \sum_{i,j}\log\left(\lambda - \sum_{k=1}^{5} v_k d_{ik}\right),$$

$$\text{subject to } \lambda - \sum_{k=1}^{5} v_k d_{ik} > 0 \ \text{ for each gene } i. \qquad \text{Eq (2)}$$

The first term is the loss function for learning the value of w_{ij} (for each gene i and drug j) that captures the degree of association between gene i and drug j. The second term can be viewed as a weighted *L2* regularization term that favors small values on w_{ij} (which improves generalizability of the learned model) with a different strength for each gene depending on the value of $\left(\lambda - \sum_{k=1}^{5} v_k d_{ik}\right)$. A gene i with a high value of $\left(\sum_{k=1}^{5} v_k d_{ik}\right)$ (i.e., MERGE score) would be regularized weakly and more inclined to have a weight value w_{ij} with a large magnitude. The last term requires the regularization parameter $\left(\lambda - \sum_{k=1}^{5} v_k d_{ik}\right)$ to be positive and encourages it to take on relatively larger values.

We iteratively estimate the optimization variables **W** and **V** using a block coordinate descent procedure[14–16] until convergence. The objective function of MERGE is not jointly convex with respect to **W** and **V**, though it is convex with respect to each set of parameters with the other set held fixed. When **V** is held fixed, the objective is convex with respect to **W**; when **W** is held fixed, the objective is convex with respect to **V**. This means that each learning step in the block coordinate descent algorithm (learning an element in **V** or in **W**) is a convex optimization problem with a single local minimum that is also the global minimum.

We performed our MERGE runs using R (version 3.3.2) on a machine with an Intel(R) Xeon(R) E5645 2.40GHz CPU and 24GB RAM. A MERGE run on the data with ~17K genes, 53 drugs and 30 samples took 12 seconds on that machine.

No modifications or improvements on the algorithm were made based on any validation analyses, i.e., the cross-validation tests involving two groups of samples, the leave-one-out cross-validation (LOOCV) test for prediction, and testing on 14 AML cell lines or the additional 12 patient samples. In each of these experiments, we chose the value of the hyperparameter λ by cross-validation (**Supplementary Note 15**).

Supplementary Note 9: Clinical information on the validation data from additional 12 AML patient samples

The additional 12 patients we used for validation (**Fig. 3c**) were enrolled in an open clinical trial (High Throughput Drug Sensitivity Assay and Genomics-Guided Treatment of Patients with Relapsed or Refractory Acute Leukemia NCT02551718). For those 12 samples, we measured gene expression levels using the newer RNA-seq technology while we had the microarray gene expression from the initial 30 patient samples (**Supplementary Note 1**). The median age was 58, and 5 had antecedent hematologic disorders. According to European LeukemiaNet criteria[4], 7 samples were in favorable risk group, 3 in intermediate-1, 1 in intermediate-2, and 8 were in the adverse risk cytogenetics group. Four samples were primary refractory, and 6 samples relapsed after allogeneic transplant. The average number of prior regimens used was 5.

Supplementary Note 10: Details on the implementations of the methods compared to MERGE

For ElasticNet, we used the R package *glmnet*[17] available on CRAN. For multi-task learning[18], the MATLAB implementation was available from Pong, Tseng, Ji, & Ye (2010) (on http://www.mypolyuweb.hk/~tkpong/). For Bayesian multi-task MKL, we used the R code provided as supplementary to Costello et al. (2014).

As mentioned in **Supplementary Note 6**, we standardized both the predictor and response variables before applying each method, as we did for MERGE. In addition, as with MERGE, we chose the tuning parameters for each method in the comparison using LOOCV. These include the mixing parameter α and regularization parameter λ for ElasticNet, the regularization parameter μ for multi-task learning, and α and β, which are, respectively, the shape and rate parameters of the Gamma priors, for Bayesian multi-task MKL.

Comparing MERGE to the other methods in **Fig. 3** and **4c** requires ordering of the gene-drug associations by the other methods, as with MERGE. For Pearson's P-value or Spearman P-value methods, we sorted the gene-drug pairs based on decreasing significance of correlation (Pearson's or Spearman correlation, depending on the method). ElasticNet and multi-task learning are regression methods, and they learn coefficients for the predictor-response variable pairs. For ElasticNet, we ran the method for each of the 53 drugs separately and concatenated the resulting gene coefficients to get a matrix of size $\#genes \times \#drugs$. The multi-task learning method, on the other hand, learns the coefficients for all drugs jointly; thus, a single run of this algorithm gives us a coefficient matrix of size $\#genes \times \#drugs$. After determining a coefficient matrix, we sorted gene-drug pairs, for each of ElasticNet and multi-task learning methods, based on decreasing absolute value of the coefficients (i.e., decreasing strength of gene-drug associations). After sorting gene-drug pairs for each method in comparison to MERGE, we incremented the number of considered top gene-drug pairs by 53 (the number of drugs per gene in our application) for each increment of 1 in the x-axis in **Fig. 3** and **4c**.

To generate heat maps for the alternative methods (**Supplementary Fig. 3**), we computed gene scores (as with MERGE scores) based on each method. For this purpose, we first sorted the gene-drug pairs in *increasing* order of their importance for each method (i.e., in increasing significance of correlation for the Pearson's and Spearman P-value methods, and in increasing absolute value of the coefficients for the ElasticNet and multi-task learning methods). For each gene G, the score was computed as the sum over all indices corresponding to that gene's associations in the sorted weight matrix. If a gene had many drug associations with high absolute weights, then those associations would be positioned towards the end of the sorted weight matrix, which would increase the score of G.

For the Bayesian multi-task MKL method, we used only gene-set views and the discretized view[20] computed on gene expression data since we had no epigenomic or proteomic profiling data from the 30 AML patient samples.

Supplementary Note 11: Computing the significance of the enrichment of the drug classes based on mechanism of action within drug clusters showing similar patient responses

To verify that drugs with similar mechanisms of action indeed showed similar response across patients, we used the dendrogram (**Fig. 4a**) generated by agglomerative hierarchical clustering. For hierarchical

clustering, we used Euclidean distance as the dissimilarity metric, and we used average linkage as the clustering method to cluster the drugs based on the response of 30 patient samples to those drugs.

In the dendrogram, for each internal node that had 3 to 8 drugs in its leaves, we first retrieved a unique set of drug classes that contained at least one drug from the node's downstream drugs. Then, we checked the enrichment of each of these drug classes in the set of the downstream drugs of that node using Fisher's exact test. Each of the enrichment p-values we present in **Fig. 4a** is FDR-corrected for the number of drug classes tested for the corresponding node using the Benjamini & Hochberg (1995) method.

Supplementary Note 12: Computing each gene's drug class specificity (DCS) measure

First, for each pair of [gene A, class B], we computed the significance of the overlap between the drugs in B and the drugs gene A's expression level is significantly associated with, measured by Fisher's exact test p-value. Then, for each gene A, we computed a specificity measure that we named *drug class specificity* (DCS) score, by combining the p-values across all classes, as follows:

$$\mathrm{DCS_A} = \frac{1}{\text{total \# of drug classes}} \sum_{B \in \{\text{drug classes}\}} -\log_{10}\left[\text{Fisher's exact test p-value}_{AB}\right] .$$

Mathematically, Fisher's exact test is based on the following numbers for each pair of [gene A, class B]: (1) the total number of drugs with which A is significantly associated, (2) the number of drugs in class B with which A is significantly associated, (3) the number of drugs in class B, and (4) the total number of drugs (53 in our experiments). The p-value measures the significance of (2) based on the hypergeometric distribution. Therefore, it measures the specificity of the association between gene A and class B.

Supplementary Note 13: Summary description of the MERGE probabilistic model

MERGE is a probabilistic, model-based approach that uses MAP to estimate parameters. A probabilistic, model-based approach provides an expressive model to describe relationships among variables. Moreover, the probabilistic relationships can be read from the learned model and thus, often directly lead to a comprehensive biological interpretation. In MERGE, the prior variance of the gene-drug weights is interpreted as the corresponding gene's biomarker potential.

We modeled W and V in a Bayesian sense (i.e., they are parameters for which belief in their values is modeled), where W is modeled as a Gaussian random variable whose variance is modeled based on V, and V is modeled as a uniform random variable (i.e., $P(V)$ is constant).

Instead of using a traditional Bayesian approach (i.e., estimating $P(\theta \mid D)$, the full posterior distribution, over parameters θ where D represents the data), we employed *maximum a posteriori probability (MAP) estimation*, i.e., obtaining a point estimate of θ that maximizes $P(\theta \mid D)$. In other words, we optimized $\log P(X, Y, W, V)$ with respect to W and V. MAP estimation has two advantages over the traditional Bayesian approach. First, estimating specific parameter values makes biological interpretation straightforward. For example, specific parameter values of v_k coefficients enable an

efficient computation of MERGE scores and straightforward interpretation of how driver features affect the MERGE score (i.e., biomarker potential). Second, MAP estimation allows a much simpler parameter learning procedure, especially when $P(\theta \mid D)$ does not have a closed-form solution. Penalized linear regression models, such as LASSO (or Ridge), also employ MAP estimation for a probabilistic model (specifically, linear regression model) with a Laplacian (or Gaussian) prior for $P(\theta)$, where the parameter θ means the W values. MERGE extends the penalized linear regression models by explicitly modeling the variance of the W parameters based on V and the driver features of genes.

Supplementary Note 14: Initialization of MERGE parameters and identifiability of the MERGE model

In our application of MERGE, we initialized all five v_k values to zero, which provided an unbiased starting point (i.e., giving an equal prior variance to the weight values of all genes).

As noted previously, the MERGE objective function represented in Eq (2) is non-convex; thus, different initializations of the V vector (of v_k values, each for a different driver feature) may lead to different learned parameters, i.e., different local minima of the objective function. In practice, however, depending on the objective function and the input data, it is possible that a roughly unique solution can be empirically identified. One way to check is to try multiple runs with different parameter initializations and see whether these runs converge to roughly the same point.

We observed that when we tried different initializations of V (and correspondingly W), the learned parameters were very similar to each other. Below, we describe our results on the consistency between the zero and random initializations.

We performed 20 different runs of MERGE where we initialized v_k values so they could be generated randomly from a standard normal distribution. Then, we compared the resulting training objective function values and the MERGE scores from these 20 runs to those from the MERGE run used in our paper (i.e., where we initialized all five v_k values to zero). We performed this experiment with the same hyperparameter value selected by LOOCV and used for the final model ($\lambda = 20$) (**Supplementary Fig. 11a**) as well as with a different λ value ($\lambda = 50$) (**Supplementary Fig. 11b**). As shown in the top of **Supplementary Fig. 11a**, for $\lambda = 20$, only 4 of 20 runs resulted in a smaller objective function (i.e., better local optima) compared to our initialization with zero v_k values, and the difference was very small. All 20 runs with random initializations resulted in almost the same v_k values shown in **Fig. 2**, and exactly the same gene rankings as those from the zero initialization of MERGE (**Supplementary Fig. 11a**, bottom). This indicates that different random initializations converged to roughly the same point. A different value of λ (λ =50) showed consistent patterns (**Supplementary Fig. 11b**).

Supplementary Note 15: Cross-validation experiments and selection of the hyperparameter λ

In each of our experiments, the regularization parameter λ was determined via cross-validation, a standard way to choose tuning parameters. Since our sample size was low, we employed LOOCV since it provides the maximum number of training samples for each fold. Cross-validation experiments were performed when measuring the prediction accuracy on left-out test data (**Fig. 5**) besides when selecting the value of the hyperparameter (sparsity tuning parameter λ).

We performed LOOCV within the training data to choose one λ value from the 19 λ values in the wide range of [1,100]. We used LOOCV to choose the values of the tuning parameters for other methods as well: elastic net regression, the multitask learning method, and the DREAM challenge winner Bayesian multi-task MKL method.

We used cross-validation tests in three settings to select the λ value: (1) *Training the model using all 30 samples* (**Fig. 3**). We first performed LOOCV on 30 samples to choose the λ value using mean squared error (MSE), and then trained the model using all 30 samples with the chosen λ value. The test MSE from the LOOCV test on 30 samples is shown for varying λ values in **Supplementary Fig. 12**. (2) *Measuring the prediction accuracy by training the model using one 12-sample batch and testing on the other 12-sample batch* (**Fig. 5a**). We performed LOOCV tests within 12 samples in each batch to choose the value of λ. We used rank correlation between the actual and predicted responses as the evaluation metric. Then we trained the model using the selected λ value in each batch and tested the prediction performance on the other batch. (3) *Measuring the prediction accuracy via LOOCV (***Fig. 5b**). In each fold for which we omitted one sample and used the remaining (n-1) samples to train the model, we performed the "inner loop" LOOCV using those (n-1) samples to choose the λ value and trained the model using (n-1) samples with the chosen λ value. We used rank correlation between the actual and predicted responses as the evaluation metric.

Supplementary Figures

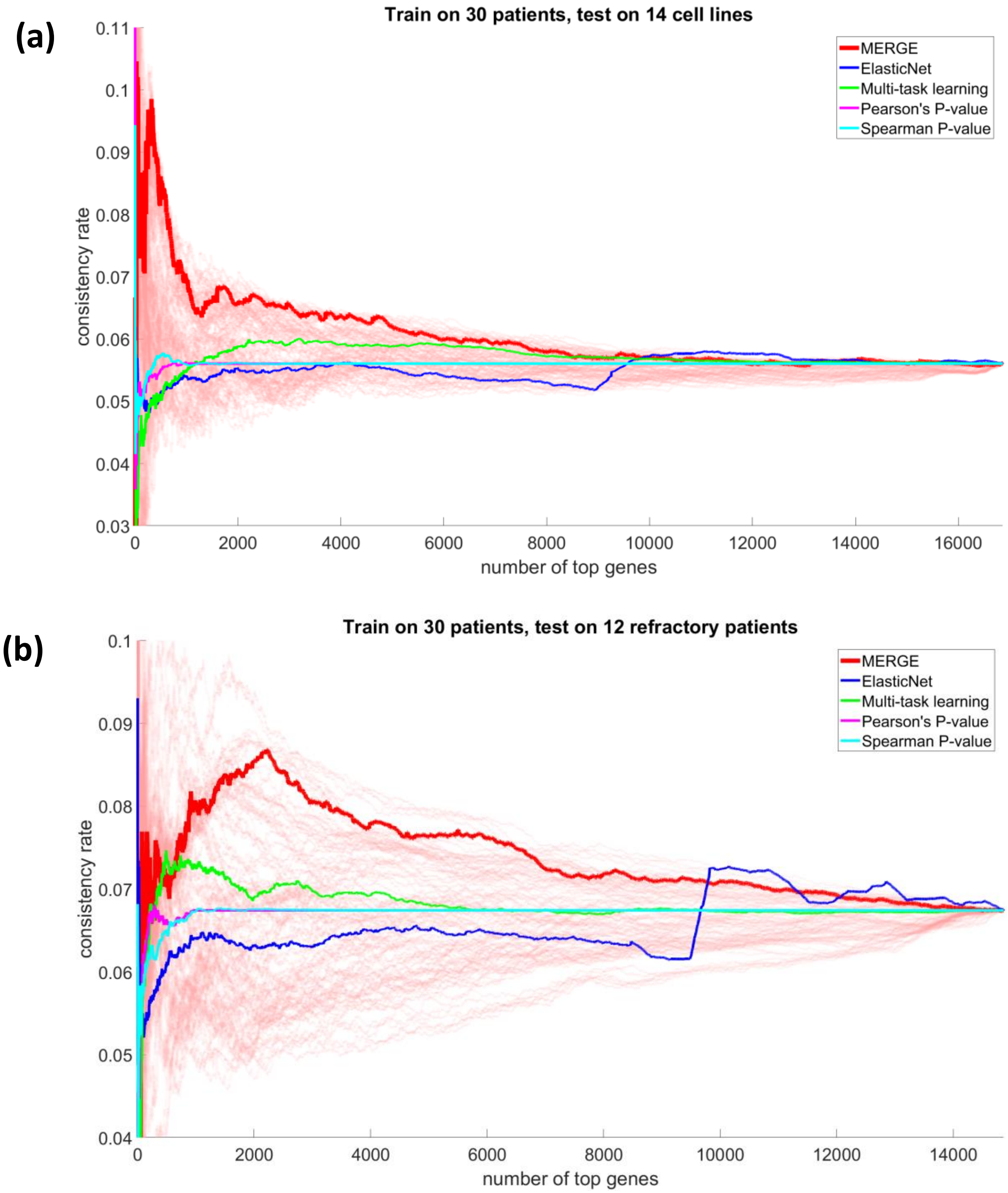

Supplementary Figure 1: Comparison of MERGE and four alternative methods to the 100 MERGE runs on random data permutations in terms of the percentage of the significant associations replicated in the left-out test data. Feature consistency achieved by 100 MERGE runs (light red) each of which uses different permutations of the training samples is compared to the consistency achieved by the actual MERGE run that uses the original training data as well as to the alternative four methods as in **Fig. 3**. We discovered gene-drug associations within the data from 30 patient samples, and tested on **(a)** the 14 cell line samples, and **(b)** additional 12 refractory patient samples.

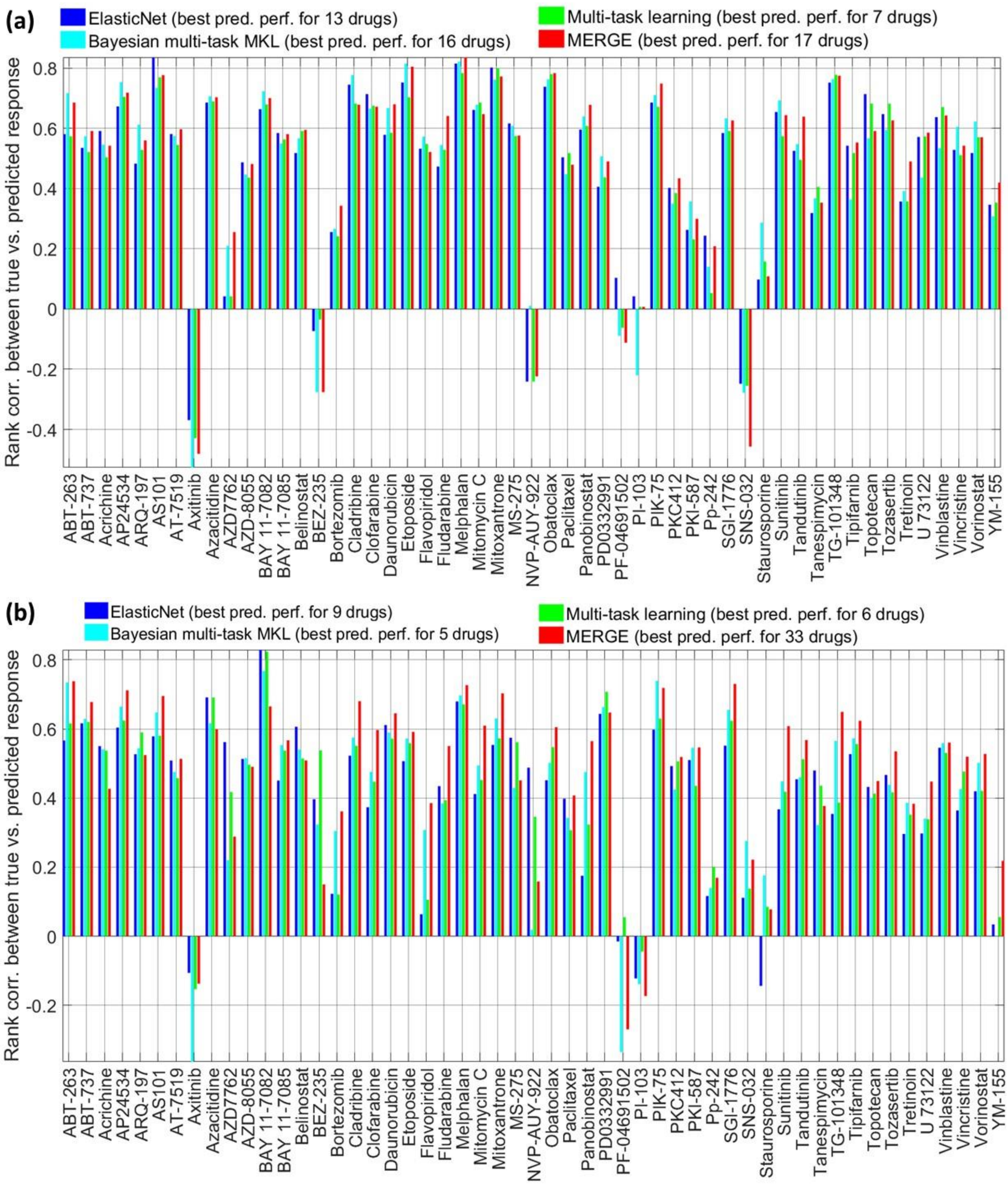

Supplementary Figure 2: Detailed view of the prediction performance of MERGE and the three alternative methods in each of the two settings shown in Fig. 5. The performance (y-axis) is measured by the rank-based (Spearman) correlation between the predicted response and actual drug response in **(a)** split test, and **(b)** LOOCV. The numbers inside the parentheses in the figure's legend (bottom right) represent the number of drugs for which the corresponding method achieved the best prediction performance.

(a) Genes identified by ElasticNet

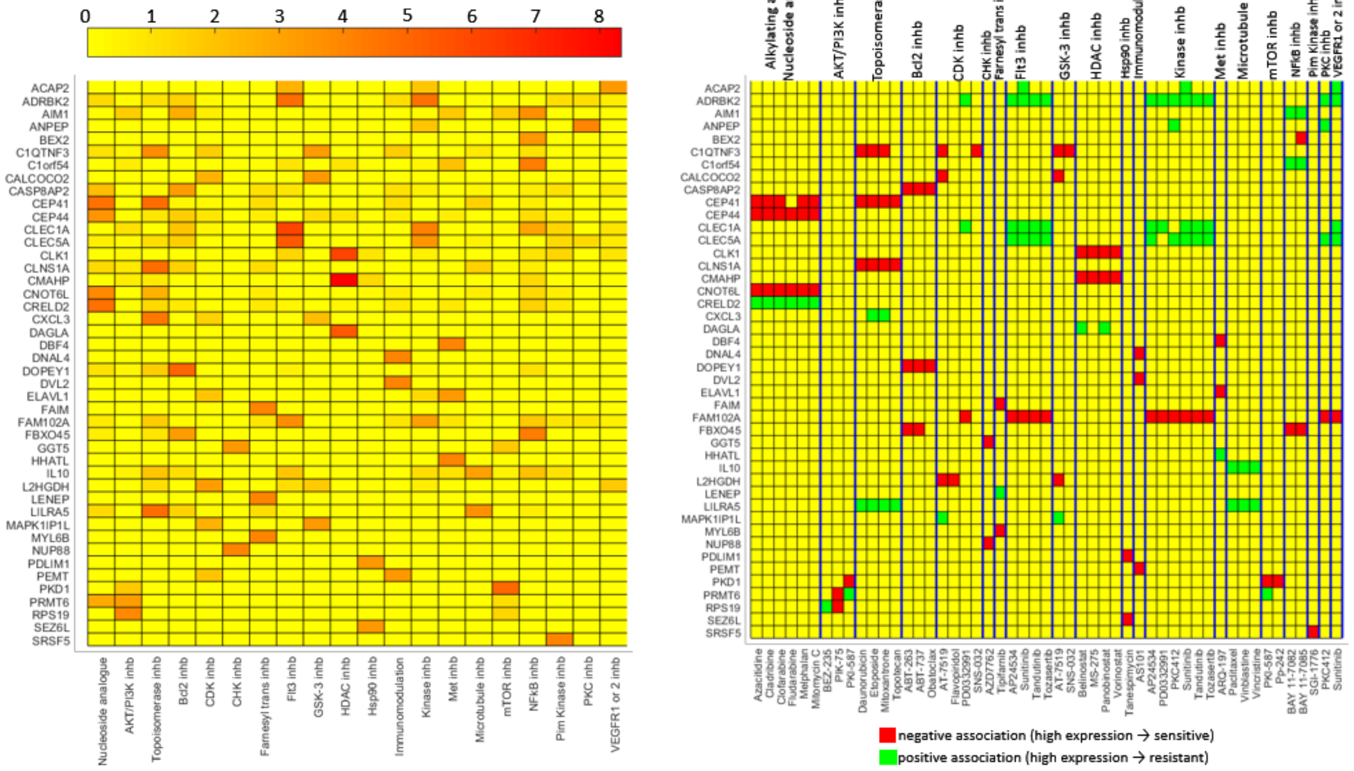

(b) Genes identified by multi-task learning

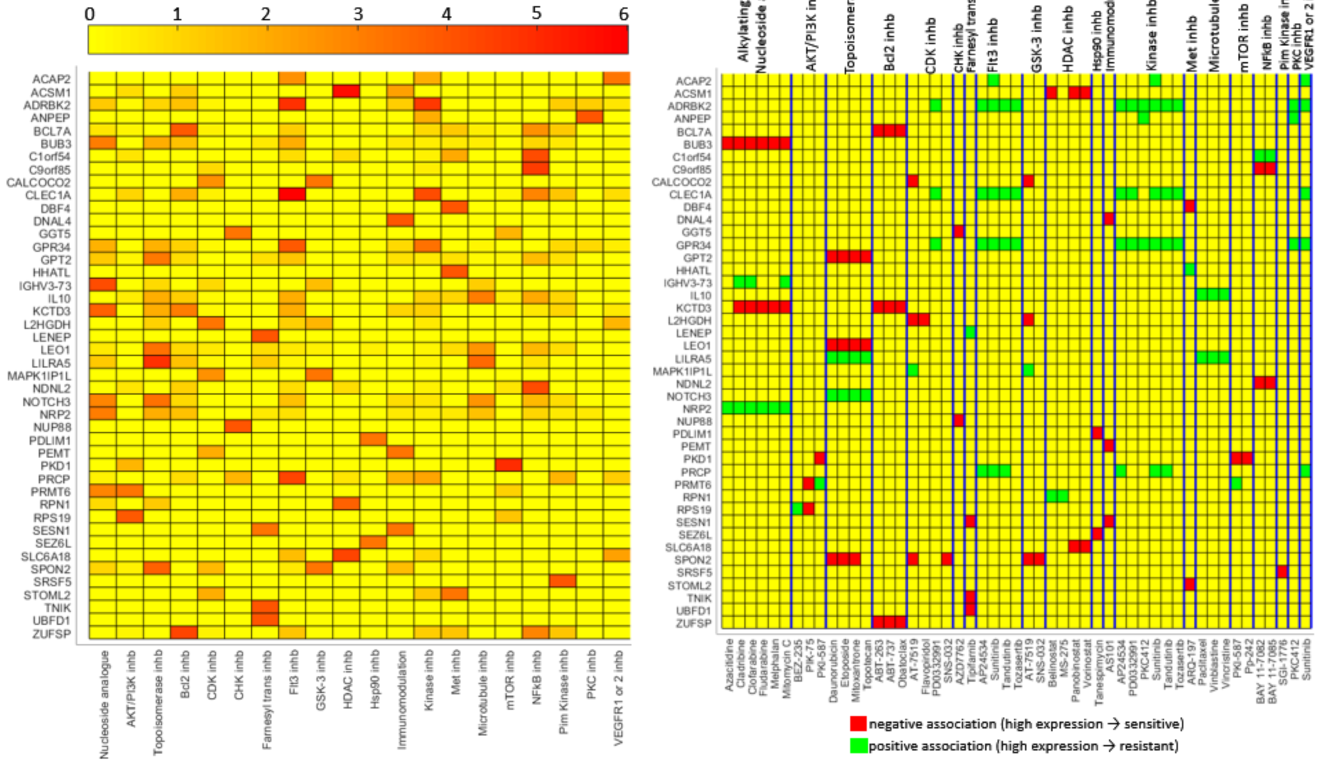

(c) Genes identified by Pearson's P-value

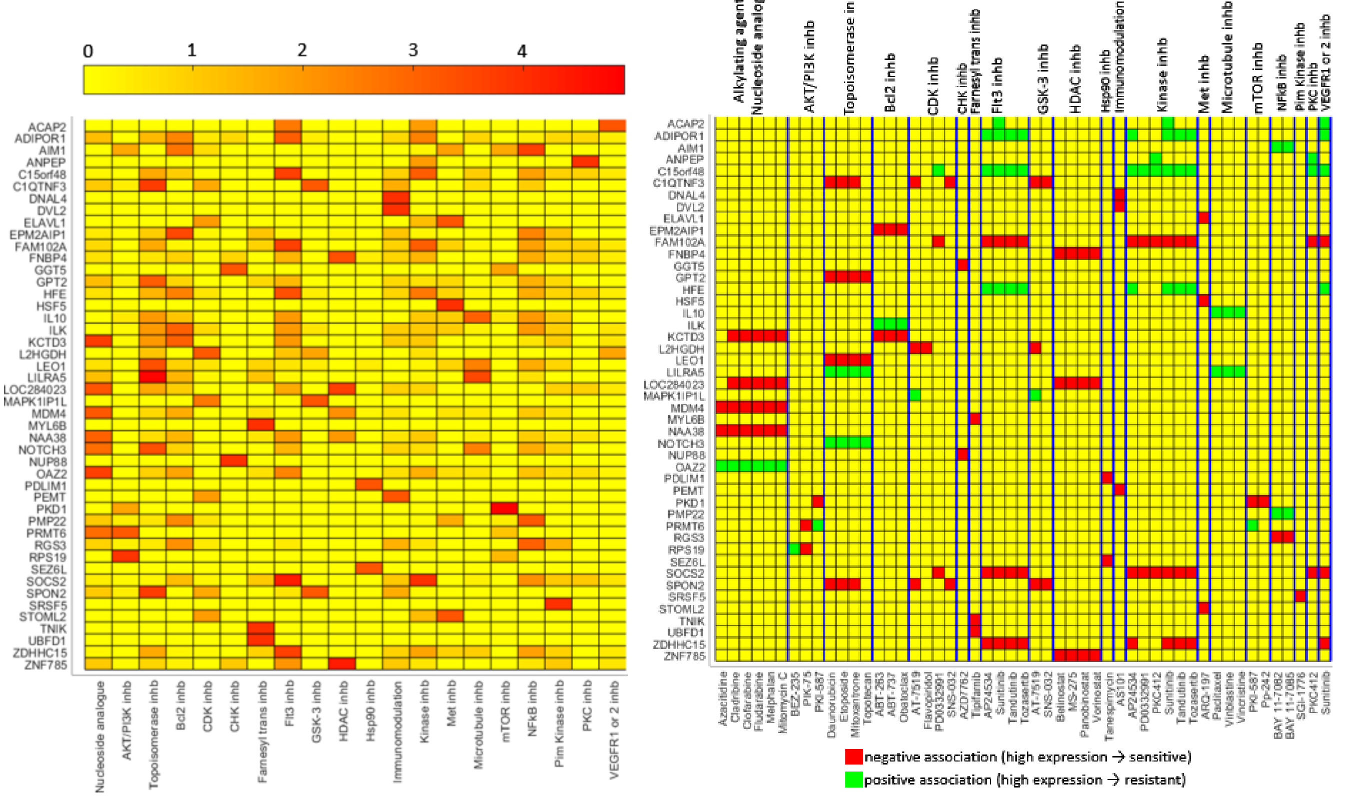

1

(d) Genes identified by Spearman P-value

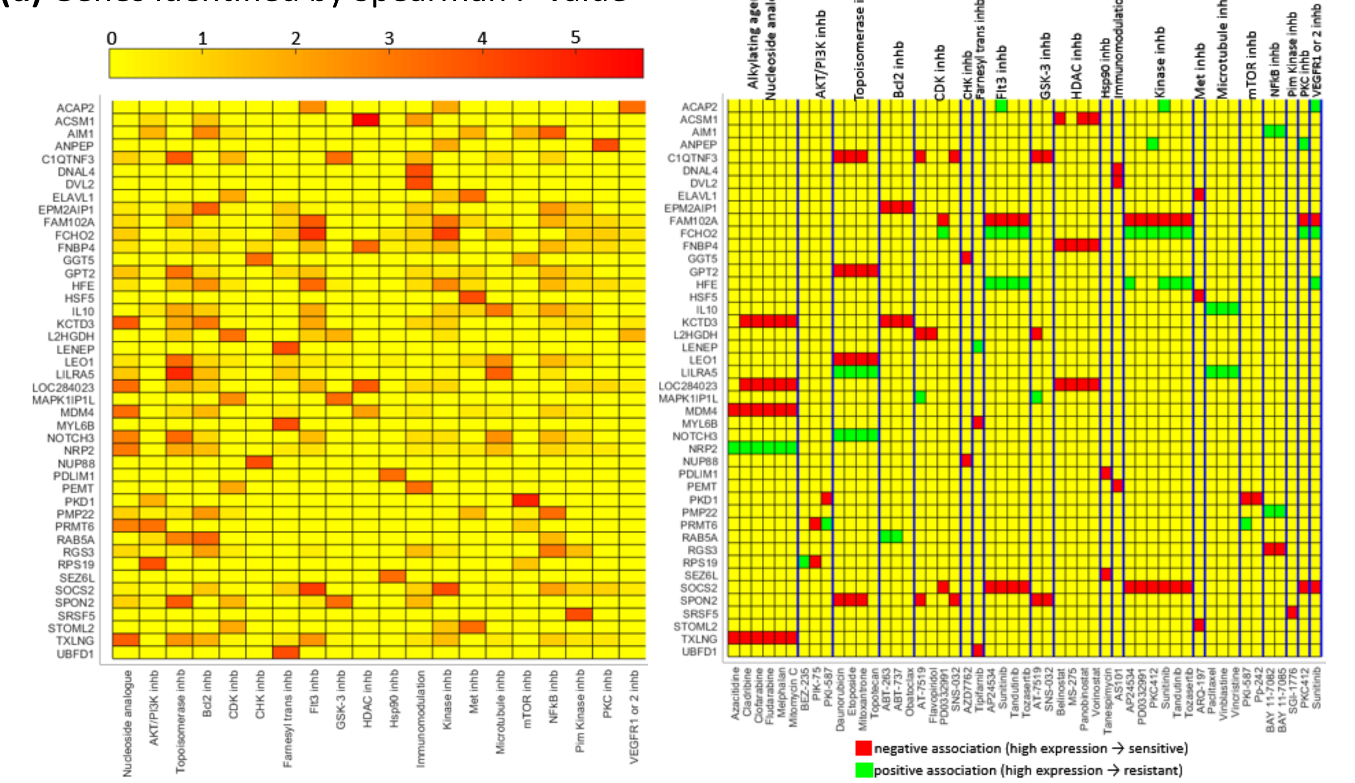

2

Supplementary Figure 3: Heat maps that correspond to Fig. 6a-c for the four alternative methods (shown in Fig. 3 and Fig. 4c). (a) ElasticNet, **(b)** Multi-task learning, (**c)** Pearson's P-value, and **(d)** Spearman P-value. In (a)-(d), the heat map on the left shows the level of specificity of each gene to each drug class, measured by $-\log_{10}[\text{Fisher's exact test p-value}]$ for the top 3 genes selected using the corresponding method; the right heat map shows the gene-drug association for the genes and drug classes shown on the left heat map.

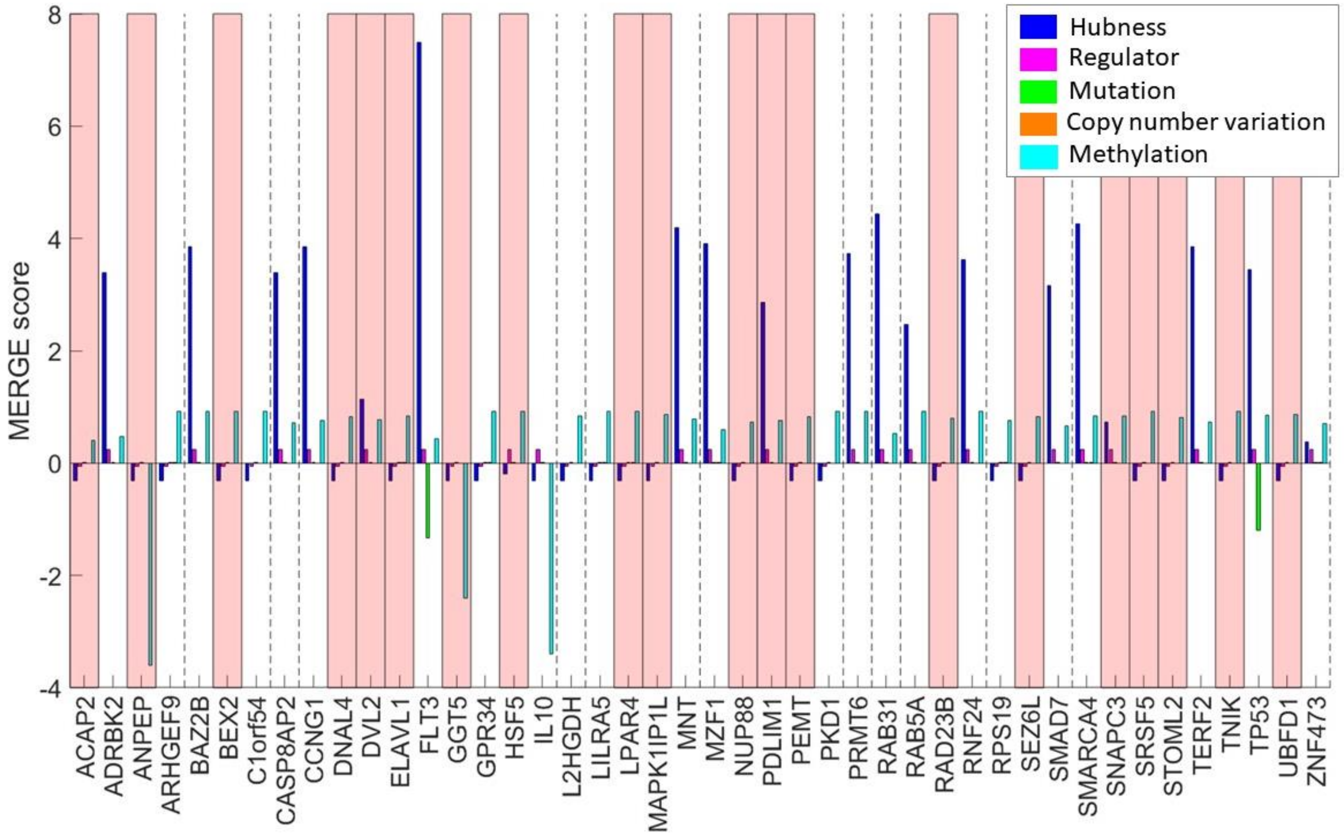

Supplementary Figure 4: For each of the genes in Fig. 6a and b, the amount of contribution of each driver feature on the MERGE score, measured by [driver feature weight × driver feature value] for a driver feature. The genes highlighted in red are associated with a single drug (i.e., the genes corresponding to the rows in **Fig. 6b** that have a single unique red or green square). We note that the genes with a high hubness contribution (blue bar > 3) tend to have significant associations with more than one drug (Fisher's exact test p-value = 1.7×10^{-5}).

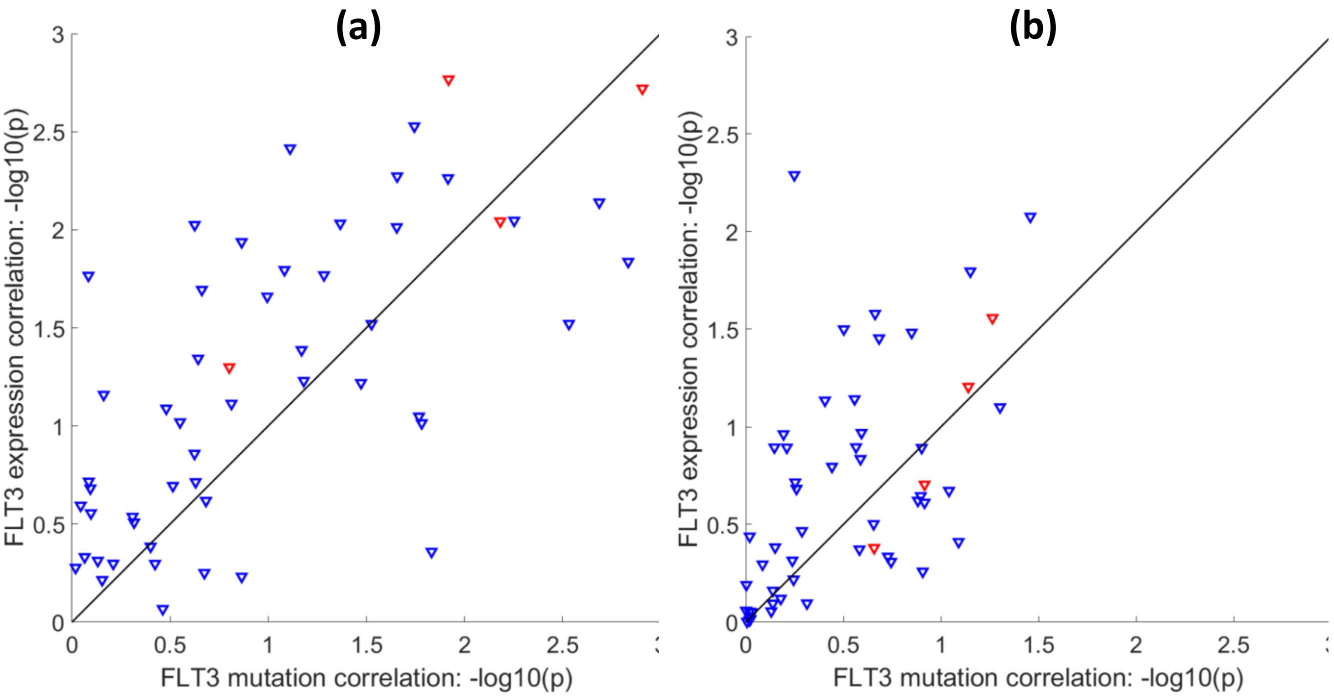

Supplementary Figure 5: Comparison of *FLT3* expression level to *FLT3* mutation status in terms of significance of correlation with drug response. For each of the 53 drugs, the $-\log_{10}$p-value representing the degrees of association between the drug response and *FLT3* expression level (y-axis) vs. *FLT3* mutation status (x-axis) is shown for **(a)** patients and **(b)** cell lines. The drugs highlighted in red on each scatter plot correspond to the four *FLT3* inhibitors (AP24534, Sunitinib, Tandutinib, Tozasertib) in the set of 53 drugs that we studied.

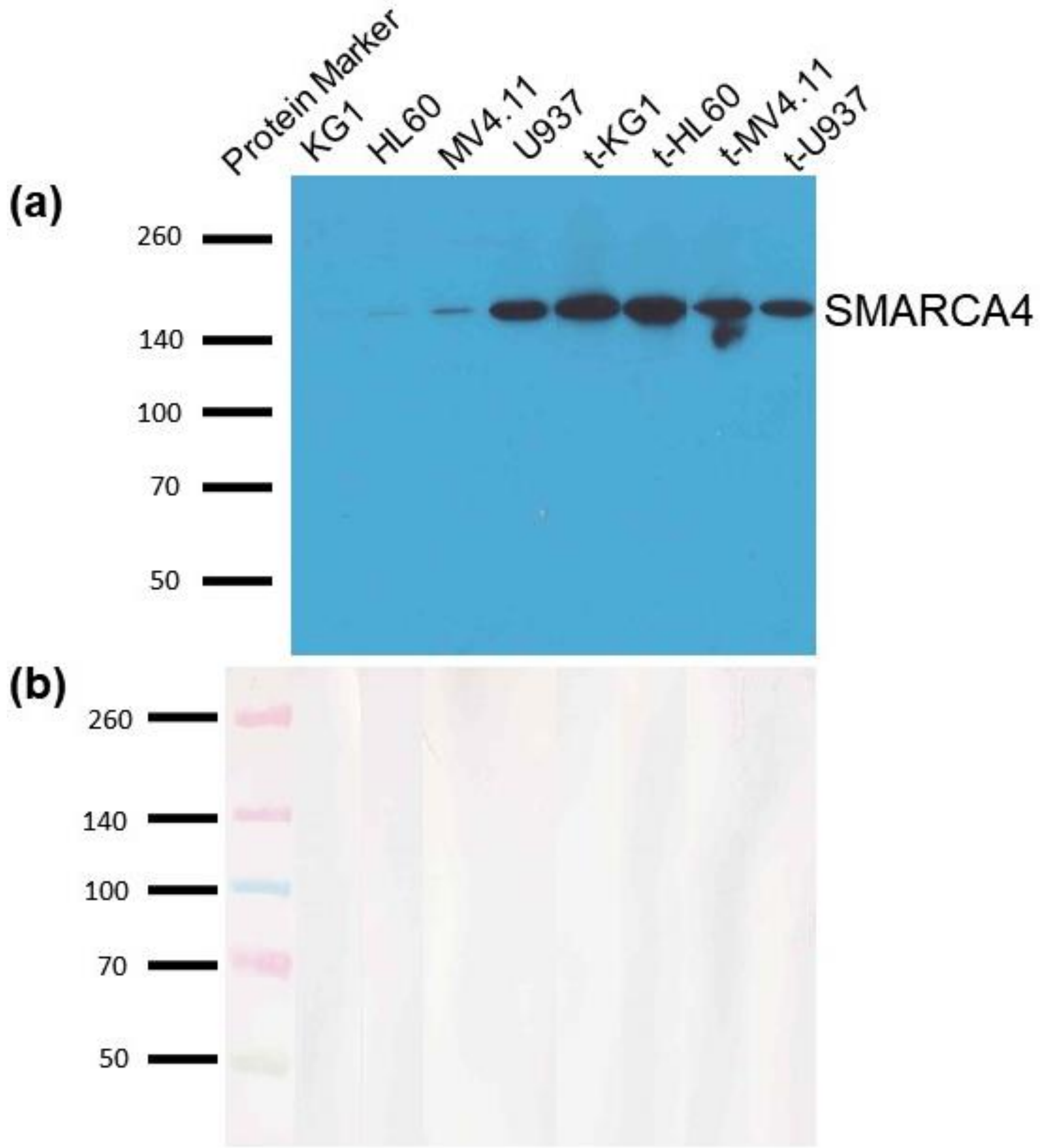

Supplementary Figure 6: Uncropped image of the Western blot of control and *SMARCA4* plasmid transfected AML cell lines: KG1, U937, HL60, and MV4.11. (a) Film. **(b)** Nitrocellulose membrane.

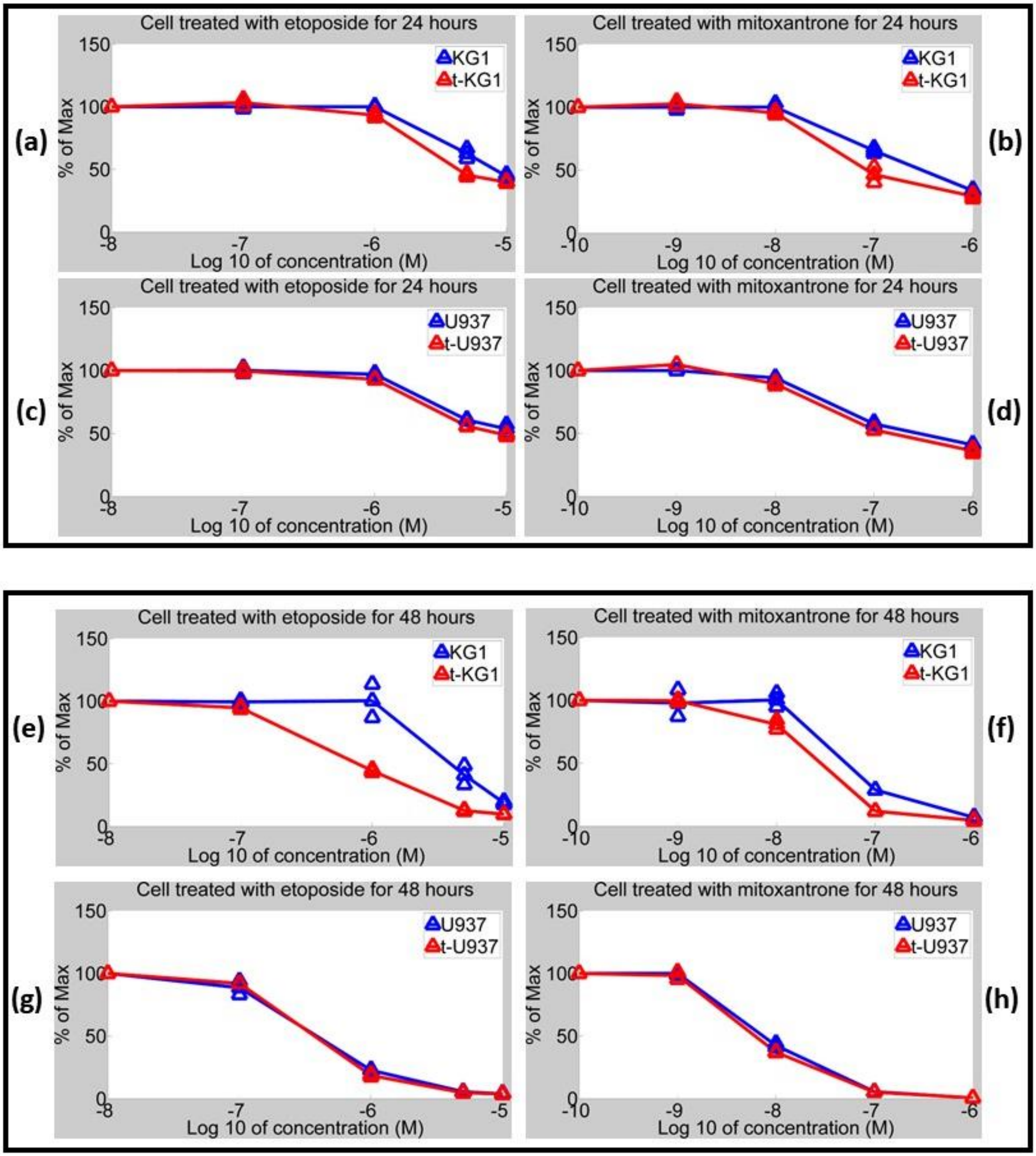

Supplementary Figure 7: The dose-response curves for cell lines treated with etoposide (left panel) and mitoxantrone (right panel) after 24 hours (a)-(d) and 48 hours (e)-(h). Each plot compares KG1 with transfected KG1 in **(a)-(b)** and **(e)-(f)**, and U937 and transfected U937 in **(c)-(d)** and **(g)-(h)**. Triangular marks indicate individual data points in duplicates and the average among them. Lines connect averages of duplicates in each concentration measured.

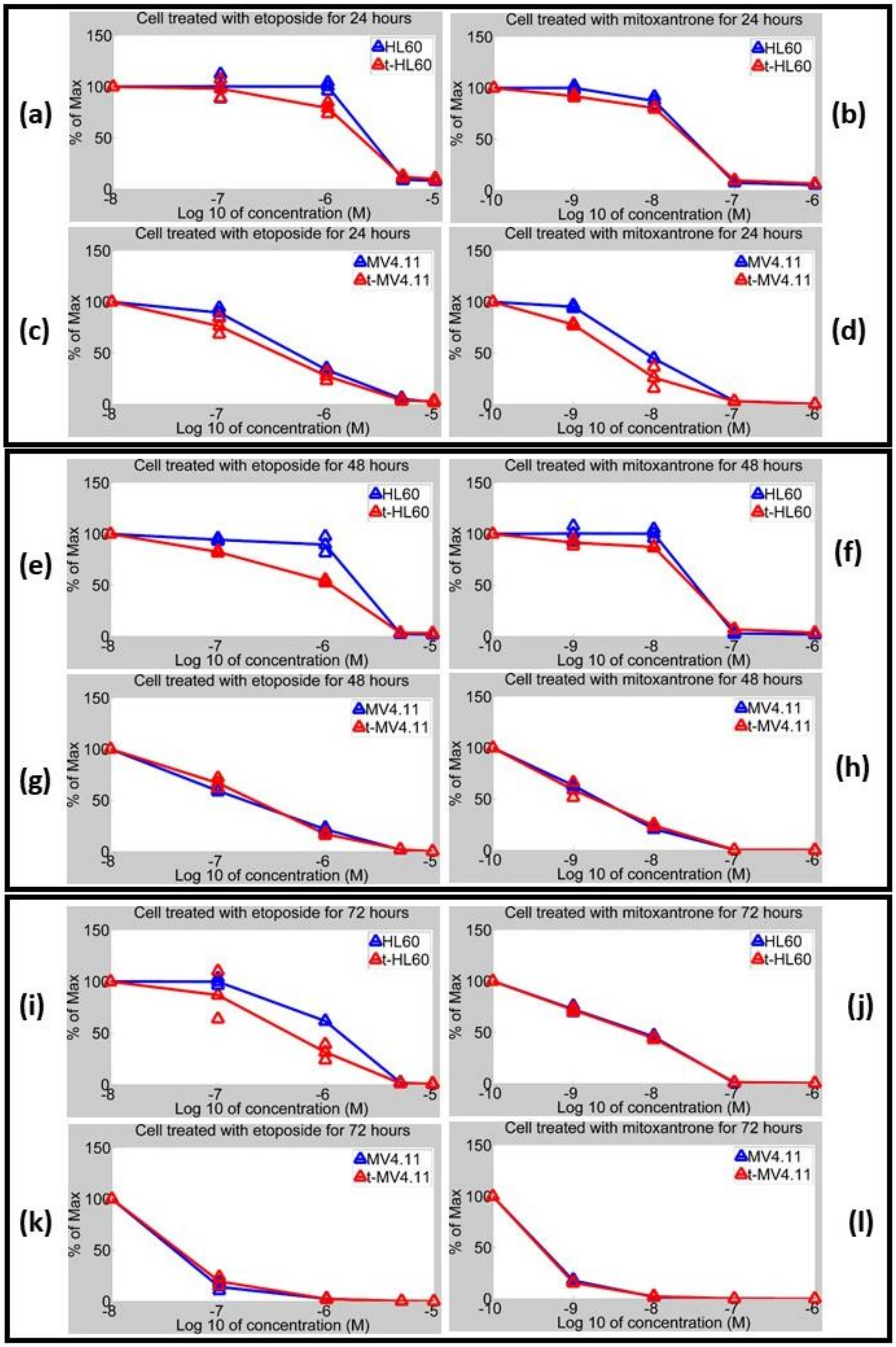

Supplementary Figure 8: The dose-response curves for cell lines treated with etoposide (left panel) and mitoxantrone (right panel) after 24 hours (a)-(d), 48 hours (e)-(h), and 72 hours (i)-(l). Each plot compares HL60 with transfected HL60 in **(a)-(b)**, **(e)-(f)**, and **(i)-(j)** and MV4.11 and transfected MV4.11 in **(c)-(d)**, **(g)-(h)**, and **(k)-(l)**. Triangular marks indicate individual data points in duplicates and the average over them. Lines connect averages over duplicates in each concentration measured.

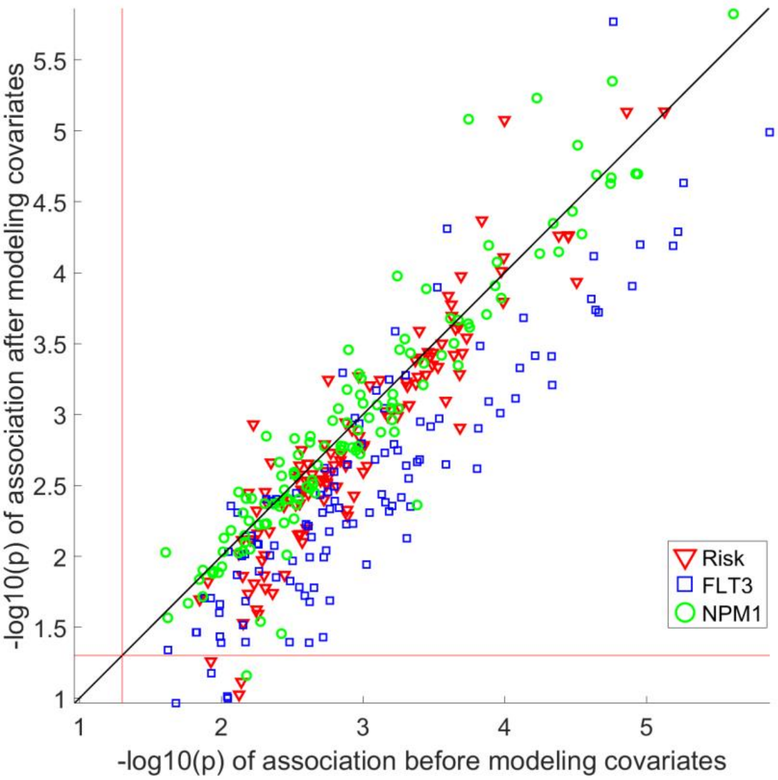

Supplementary Figure 9: Comparison of the significance of the gene-drug associations ($-\log_{10}$ p-value) with (y-axis) vs. without (x-axis) adding the available risk group/cytogenetic features as covariates. Red corresponds to cytogenetic risk covariate, blue corresponds to *FLT3* mutation status, and green corresponds to *NPM1* mutation status. The red vertical and horizontal lines correspond to $-\log_{10} 0.05$. Each dot corresponds to one of the 119 unique gene-drug associations shown as red or green on the heat map in **Fig. 6b**.

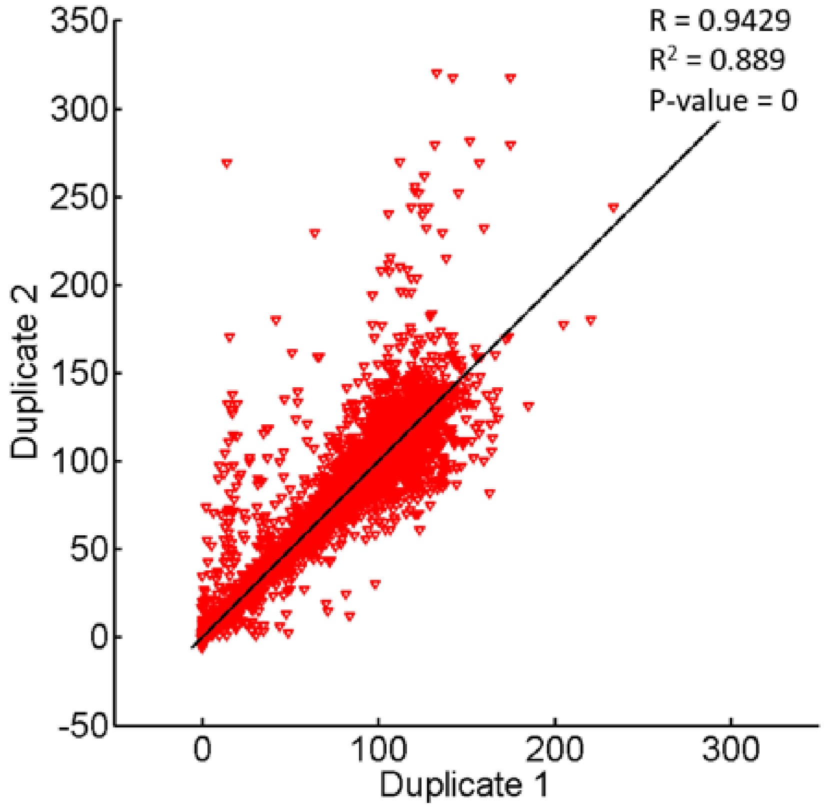

Supplementary Figure 10: Scatter plot demonstrating the reproducibility of high-throughput drug sensitivity assay. Each dot corresponds to a combination of a drug, a sample, and a specific concentration. The correlation coefficient, R^2 (squared correlation coefficient), and *p*-value are computed based on Pearson's correlation.

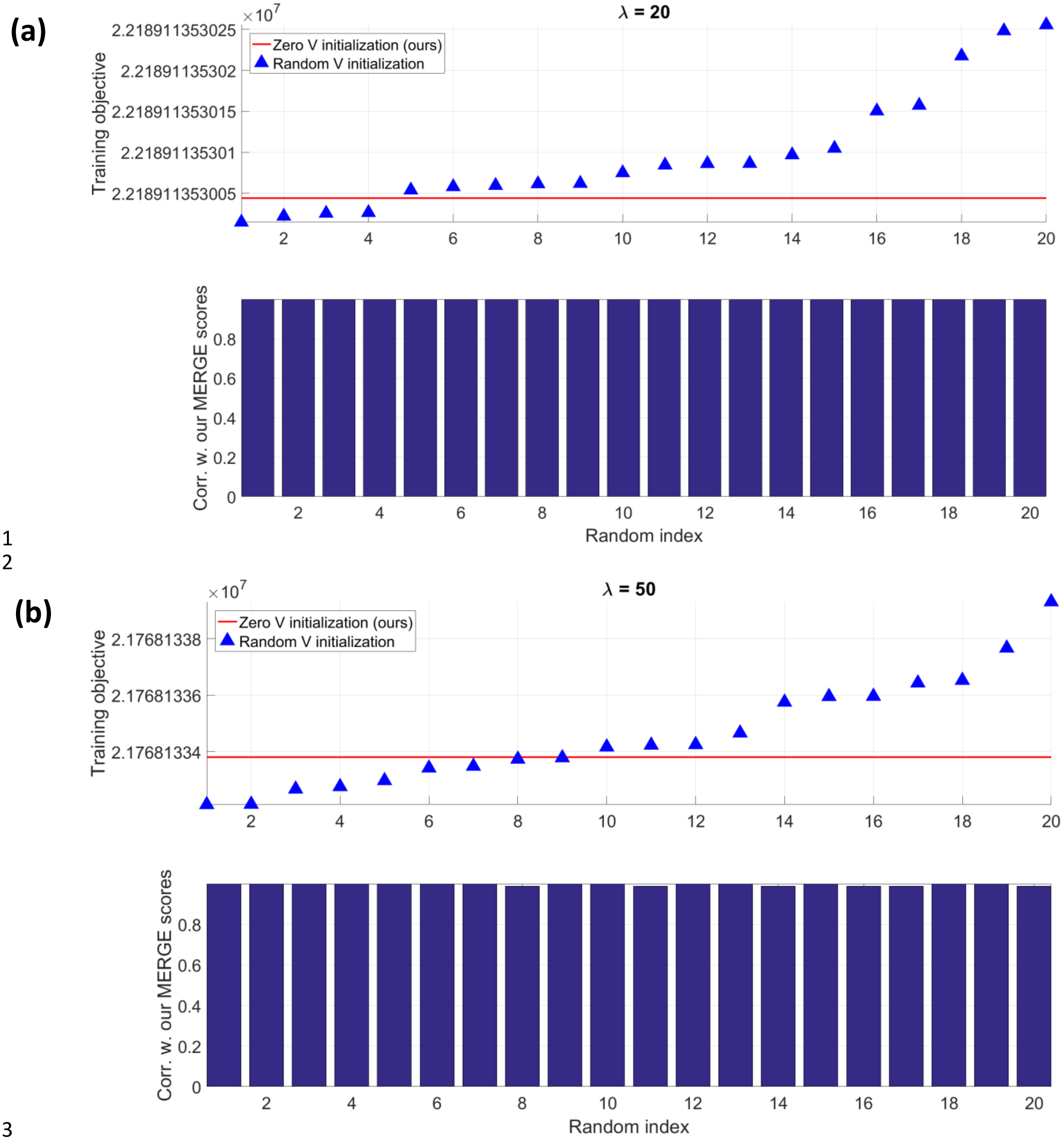

Supplementary Figure 11: For 20 random initializations of the learning parameters (x-axis), the training objective function values (top) and the Spearman correlation of the learned MERGE scores from each random initialization with those from zero initialization (used for our paper) (bottom). (a) We used the same λ value as the final MERGE model (i.e., trained based on 30 samples) that was selected by LOOCV ($\lambda = 20$). **(b)** We used an additional value for the hyperparameter $\lambda = 50$.

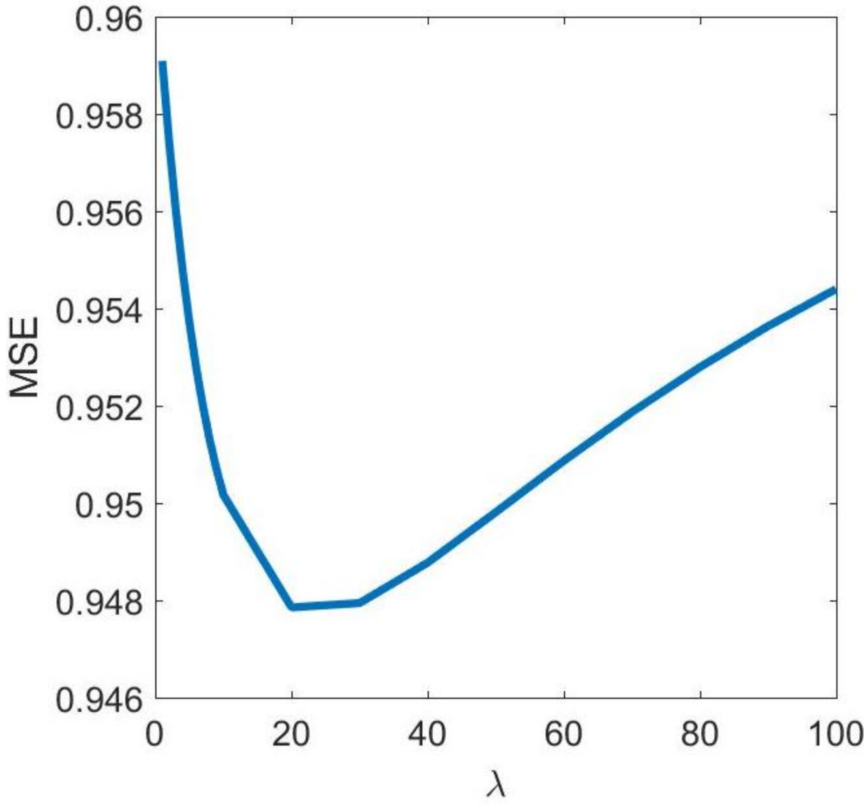

Supplementary Figure 12: The test MSE measured by the LOOCV test for varying λ values. λ value (combined with the driver features) is used to regularize weight values (w_{ij}) for gene-drug pairs. Based on this plot, in our experiment with 30 samples, we use $\lambda = 20$ since it results in the lowest test MSE.

Supplementary Tables

Supplementary Table 1: Some properties for each of the 53 drugs. p-values listed in the 4th column are FDR-corrected for the number of drugs. Since some drugs are in multiple classes, the last column shows the number of genes significantly associated with the drug for each class the drug is in. This column is empty for the 3 drugs (Acrichine, U 73122 and YM-155) assigned only to the "Other" class, which contains the drugs that do not belong to any of the 24 drug mechanism classes based on their mechanisms of action. The footnotes (a)-(k) present details of the drugs that have a "1" in the 3rd column or have a "-" in the last column. We note that for the drugs ABT-263 and ABT-737, the "1" in the 3rd column reports a successor drug that is effective in AML.

Drug Name	Reported Clinical Efficacy in AML Treatment	Whether Any Undergoing Clinical Evaluations in AML	Significance of Association of AUC with CR	#Significantly Associated Genes	#Significantly Associated Genes after Cell Line Consistency Filter	#Significantly Associated Genes after Cell Line Consistency and Drug Class Specificity Filters
ABT-263	0	1[a]	0.0023	1825	142	[Bcl2 inhb: 10]
ABT-737	0	1[b]	0.0039	1727	122	[Bcl2 inhb: 11]
Acrichine	0	0	0.0267	1169	119	- [i]
AP24534 (=Ponatinib)	0	0	0.0153	1911	93	[Flt3 inhb: 11]; [Kinase inhb: 5]
ARQ-197 (=Tivantinib)	0	0	0.003	1103	73	[Met inhb: 6]
AS101	0	0	0.0086	2011	123	[Immunomodulation: 12]
AT-7519	0	0	0.4349	653	61	[CDK inhb: 1]; [GSK-3 inhb: 14]
Axitinib	0	0	0.9724	35	2	[Kinase inhb: 0]; [VEGFR1 or 2 inhb: 0]
Azacitidine	1	0	0.0379	1477	76	[Nucleoside analogue: 22]
AZD7762	0	0	0.8083	171	15	[CHK inhb: 5]
AZD-8055	0	0	0.0039	649	32	[mTOR inhb: 1]
BAY 11-7082	0	0	0.003	2568	141	[NFkB inhb: 25]
BAY 11-7085	0	0	0.0021	1554	66	[NFkB inhb: 11]
Belinostat	0	1[c]	0.0159	696	42	[HDAC inhb: 16]
BEZ-235	0	0	0.2664	138	11	[AKT/PI3K inhb: 4]
Bortezomib	1	0	0.0094	263	4	[Proteosome inhb: 0]

Drug Name	Reported Clinical Efficacy in AML Treatment	Whether Any Undergoing Clinical Evaluations in AML	Significance of Association of AUC with CR	#Significantly Associated Genes	#Significantly Associated Genes after Cell Line Consistency Filter	#Significantly Associated Genes after Cell Line Consistency and Drug Class Specificity Filters
Cladribine	1	0	0.0497	1420	169	[Nucleoside analogue: 69]
Clofarabine	1	0	0.0267	908	93	[Nucleoside analogue: 54]
Daunorubicin	1	0	0.0224	1833	73	[Topoisomerase inhb: 12]
Etoposide	1	0	0.0317	1699	78	[Topoisomerase inhb: 12]
Flavopiridol	1	0	0.6414	346	13	[CDK inhb: 2]
Fludarabine	1	0	0.0086	1169	95	[Nucleoside analogue: 42]
Melphalan	0	1[(d)]	0.0098	2323	151	[Nucleoside analogue: 45]
Mitomycin C	0	0	0.073	1428	80	[Nucleoside analogue: 33]
Mitoxantrone	1	0	0.0457	2106	90	[Topoisomerase inhb: 14]
MS-275	0	0	0.1161	679	26	[HDAC inhb: 7]
NVP-AUY-922	0	0	0.5051	121	4	[Hsp90 inhb: 2]
Obatoclax	0	1[(e)]	0.0276	1177	48	[Bcl2 inhb: 6]
Paclitaxel	0	0	0.0684	590	27	[Microtubule inhb: 4]
Panobinostat	1	0	0.0159	680	41	[HDAC inhb: 18]
PD0332991 (=Palbociclib)	0	1[(f)]	0.0023	1739	194	[CDK inhb: 0]; [Kinase inhb: 3]
PF-04691502	0	0	0.9764	63	1	[AKT/PI3K inhb: 0]; [mTOR inhb: 0]
PI-103	0	0	0.4311	47	0	[AKT/PI3K inhb: 0]; [mTOR inhb: 0]
PIK-75	0	0	0.0171	2151	103	[AKT/PI3K inhb: 1]
PKC412 (=Midostaurin)	1	0	0.0344	794	38	[Kinase inhb: 4]; [PKC inhb: 1]

Drug Name	Reported Clinical Efficacy in AML Treatment	Whether Any Undergoing Clinical Evaluations in AML	Significance of Association of AUC with CR	#Significantly Associated Genes	#Significantly Associated Genes after Cell Line Consistency Filter	#Significantly Associated Genes after Cell Line Consistency and Drug Class Specificity Filters
PKI-587 (=Gedatolisib)	0	1[(g)]	0.0385	677	86	[AKT/PI3K inhb: 1]; [mTOR inhb: 1]
Pp-242	0	0	0.868	179	11	[mTOR inhb: 0]
SGI-1776	0	0	0.0039	1758	95	[Pim Kinase inhb: 2]
SNS-032	0	0	0.3771	178	15	[CDK inhb: 1]; [GSK-3 inhb: 4]
Staurosporine	0	0	0.3771	83	11	[Kinase inhb: 1]
Sunitinib	0	1[(h)]	0.0344	1045	48	[Flt3 inhb: 9]; [Kinase inhb: 4]; [VEGFR1 or 2 inhb: 1]
Tandutinib	0	0	0.0053	961	43	[Flt3 inhb: 11]; [Kinase inhb: 8]
Tanespimycin	0	0	0.2049	459	28	[Hsp90 inhb: 6]
TG-101348	0	0	0.0344	1602	90	[JAK/STAT inhb: 1]
Tipifarnib	1	0	0.1329	1592	67	[Farnesyl trans inhb: 9]
Topotecan	1	0	0.1088	856	38	[Topoisomerase inhb: 10]
Tozasertib	0	0	0.0239	984	46	[Aurora kinase inhb: 0]; [Flt3 inhb: 9]; [Kinase inhb: 6]
Tretinoin	1	0	0.0039	534	69	[Retinoid: 4]
U 73122	0	0	0.0344	679	36	-[(j)]
Vinblastine	0	0	0.07	1469	180	[Microtubule inhb: 10]
Vincristine	0	0	0.0874	1154	102	[Microtubule inhb: 8]
Vorinostat	1	0	0.0171	621	29	[HDAC inhb: 15]
YM-155	0	0	0.3771	249	9	-[(k)]

(a) Its successor Bcl2 inhibitor ABT199 is effective in AML.
(b) Its successor Bcl2 inhibitor ABT199 is effective in AML.
(c) It has been tested in phase II trial in AML as a single agent.
(d) It is one of the 2 drugs of the fludarabine-melphalan preparative regimen for allogeneic transplant that has been used frequently in AML.

(e) It has been studied in a phase I/II clinical trial as a single agent in AML.
(f) It is undergoing clinical trial in AML.
(g) It is undergoing clinical trial in AML.
(h) It has been studied in a phase I/II clinical trial in AML with chemotherapy.
(i) It is an antimalarial drug in the "Other" class.
(j) It is a phospholipase inhibitor in the "Other" class.
(k) It is a survivin suppressant in the "Other" class.

Supplementary Table 2: Top 10 MERGE-scoring genes in each drug class shown in Fig. 6a. Some drug classes have less than 10 genes that are specifically associated with that class and whose associations are conserved in CCLE data. Column 2 (#Drugs) indicates the number of drugs in the corresponding drug class. Column 3 (#Genes) indicates the number of top MERGE genes specifically associated with the corresponding class (the full list is in **Supplementary Data 6**). The classes highlighted in red contain only 1 drug, and the genes highlighted in yellow are those discussed in the main text.

Drug Class	#Drugs	#Genes	Top 10 Genes
Alkylating agent/ Nucleoside analogue	6	58	*RAB31, CCNG1, TERF2, ZNRD1, RNF113A, SMARCC1, ZNF697, MDM4, CNOT6L, ADAM28*
AKT/PI3K inhibitor	5	2	*PRMT6, RPS19*
Topoisomerase inhibitor	4	24	*SMARCA4, MZF1, TP53, LEO1, BCL2, ILF3, PRMT7, CEP41, CLNS1A, GPT2*
Bcl2 inhibitor	3	13	*CASP8AP2, SMAD7, RAB5A, ZNF420, FBXO45, ILK, BCL7A, ZUFSP, SHQ1, KCTD3*
CDK inhibitor	4	1	*L2HGDH*
CHK Inhibitor	1	2	*NUP88, GGT5*
Farnesyl transferase inhibitor	1	6	*SNAPC3, TNIK, UBFD1, MYL6B, FAIM, LENEP*
Flt3 inhibitor	4	16	*FLT3, ADRBK2, ZNF473, ZDHHC15, GPR34, ATAT1, FAM102A, FCHO2, MYH3, PITRM1*
GSK-3 inhibitor	2	10	*LPAR4, MAPK1IP1L, RAD23B, NXF3, RNASE2, TNFRSF18, CALCOCO2, RNASE3, SPON2, C1QTNF3*
HDAC inhibitor	4	25	*MNT, BAZ2B, RNF24, MZF1, ZNF785, ZNF763, RAB3D, PSMC5, CCDC167, CMAHP*
Hsp90 inhibitor	2	2	*PDLIM1, SEZ6L*
Immunomodulation, anti-inflammatory	1	4	*DVL2, PEMT, DNAL4, SESN1*
Kinase inhibitor	8	11	*FLT3, ADRBK2, GPR34, FAM102A, FCHO2, PITRM1, SRSF3, C15orf48, SOCS2, CLEC5A*
Met inhibitor	1	5	*HSF5, ELAVL1, STOML2, DBF4, HHATL*
Microtubule inhibitor	3	2	*LILRA5, IL10*
mTOR inhibitor	5	1	*PKD1*
NFkB inhibitor	2	10	*ARHGEF9, BEX2, C1orf54, AIM1, YY1AP1, NDNL2, C9orf85, GGA2, PMP22, RGS3*

Drug Class	#Drugs	#Genes	Top 10 Genes
Pim Kinase inhibitor	1	1	*SRSF5*
PKC inhibitor	1	1	*ANPEP*
VEGFR1 or 2 inhibitor	2	1	*ACAP2*

Supplementary Table 3: Whether each of the 8 expression markers identified by MERGE (rows) would have been identified by the alternative methods in Fig. 3 and 4a (columns). A checkmark means the marker in the corresponding row was identified by the method in the corresponding column. The rows of heat maps in **Fig. 6** show the entire list of genes identified by each method.

Gene	ElasticNet	Multi-task learning	Pearson's P-value	Spearman P-value
SMARCA4				
CASP8AP2	✓			
L2HGDH	✓	✓	✓	✓
FLT3				
MNT				
BAZ2B				
MZF1				
BEX2	✓			

Supplementary Table 4: IC50 and AUC values in the KG1, transfected KG1, U937 and transfected U937 cell lines.

**AUC (24 hr)**	**KG1**	**t-KG1**	**U937**	**t-U937**
Etoposide	594.318	575.157	583.107	575.956
Mitoxantrone	479.329	457.108	466.419	453.636
**AUC (48 hr)**	**KG1**	**t-KG1**	**U937**	**t-U937**
Etoposide	603.722	503.202	463.981	464.517
Mitoxantrone	417.081	358.726	312.344	308.787
**AUC (72 hr)**	**KG1**	**t-KG1**	**U937**	**t-U937**
Etoposide	602.589	462	446.642	429.982
Mitoxantrone	427.033	306.129	293.408	292.742
**IC_{50} (24 hr)**	**KG1**	**t-KG1**	**U937**	**t-U937**
Etoposide	-5.252	-5.384	-5	-5.09
Mitoxantrone	-6.95	-7.034	-6.782	-6.932
**IC_{50} (48 hr)**	**KG1**	**t-KG1**	**U937**	**t-U937**
Etoposide	-5.318	-5.948	-6.374	-6.38
Mitoxantrone	-7.052	-7.496	-8.018	-8.03
**IC_{50} (72 hr)**	**KG1**	**t-KG1**	**U937**	**t-U937**
Etoposide	-5.354	-6.308	-6.548	-6.71
Mitoxantrone	-7.088	-8	-8.102	-8.108

Supplementary References

1. Trapnell, C. *et al.* Transcript assembly and quantification by RNA-Seq reveals unannotated transcripts and isoform switching during cell differentiation. *Nat. Biotechnol.* **28,** 511–515 (2010).

2. Barretina, J. *et al.* The Cancer Cell Line Encyclopedia enables predictive modelling of anticancer drug sensitivity. *Nature* **483,** 603–7 (2012).

3. Johnson, W. E., Li, C. & Rabinovic, A. Adjusting batch effects in microarray expression data using empirical Bayes methods. *Biostatistics* **8,** 118–127 (2007).

4. Döhner, H. *et al.* Diagnosis and management of acute myeloid leukemia in adults: Recommendations from an international expert panel, on behalf of the European LeukemiaNet. *Blood* **115,** 453–474 (2010).

5. Hande, K. R., Stein, R. S., McDonough, D. A., Greco, F. A. & Wolff, S. N. Effects of high-dose cytarabine. *Clin. Pharmacol. Ther.* **31,** 669–674 (1982).

6. The Cancer Genome Atlas Research Network. Genomic and Epigenomic Landscapes of Adult De Novo Acute Myeloid Leukemia — NEJM. *N. Engl. J. Med.* **368,** 2059–2074 (2013).

7. Gentles, A. J., Plevritis, S. K., Majeti, R. & Alizadeh, A. A. Association of a leukemic stem cell gene expression signature with clinical outcomes in acute myeloid leukemia. *JAMA* **304,** 2706–15 (2010).

8. Logsdon, B. A. *et al.* Sparse expression bases in cancer reveal tumor drivers. *Nucleic Acids Res.* **43,** 1332–1344 (2015).

9. Moarii, M., Boeva, V., Vert, J.-P. & Reyal, F. Changes in correlation between promoter methylation and gene expression in cancer. *BMC Genomics* **16,** 873 (2015).

10. Tibshirani, R. Regression Selection and Shrinkage via the Lasso. *J. R. Stat. Soc. B* **58,** 267–288 (1994).

11. Tibshirani, R. The lasso method for variable selection in the cox model. *Stat. Med.* **16,** 385–395 (1997).

12. Benjamini, Y. & Hochberg, Y. Controlling the false discovery rate: a practical and powerful approach to multiple testing. *J. R. Stat. Soc. B* **57,** 289–300 (1995).

13. Koller, D. & Friedman, N. *Probabilistic Graphical Models: Principles and Techniques*. (The MIT Press, 2009).

14. Hildreth, C. A Quadratic Programming Procedure. *Nav. Res. Logist. Q.* **4,** 79–85 (1957).

15. Warga, J. Minimizing Certain Convex Functions. *SIAM J. Appl. Math.* **11,** 588–93 (1963).

16. Tseng, P. Convergence of a block coordinate descent method for nondifferentiable minimization. *J. Optim. Theory Appl.* **109,** 475–494 (2001).

17. Friedman, J., Hastie, T. & Tibshirani, R. Regularization Paths for Generalized Linear Models via

Coordinate Descent. *J. Stat. Softw.* **33,** 1–22 (2010).

18. Yuan, H., Paskov, I., Paskov, H., González, A. J. & Leslie, C. S. Multitask learning improves prediction of cancer drug sensitivity. *Sci. Rep.* **6,** 31619 (2016).

19. Pong, T. K., Tseng, P., Ji, S. & Ye, J. Trace Norm Regularization: Reformulations, Algorithms, and Multi-Task Learning. *SIAM J. Optim.* **20,** 3465–3489 (2010).

20. Costello, J. C. *et al.* A community effort to assess and improve drug sensitivity prediction algorithms. *Nat. Biotechnol.* **32,** 1–103 (2014).
