# Supplementary material for: A machine learning approach to integrate big data for precision medicine in acute myeloid leukemia
Source: Nat Commun. 2018 Jan 3;9:42. doi: 10.1038/s41467-017-02465-5 (PMC5752671; doi:10.1038/s41467-017-02465-5)
Supplement: Supplementary file 3 — Description of Additional Supplementary Files [file 41467_2017_2465_MOESM3_ESM.pdf]

Description of Additional Supplementary Files

File Name: Supplementary Data 1
Description: **Expression data and drug sensitivity measures (AUC, IC50, EC50 and Amax) from the 30 AML patient samples and 14 leukemia cell lines**: Expression data for the cell lines originated from the public CCLE database. EC50 is the concentration of a drug that gives half-maximal response. IC50 is the concentration of an inhibitor where the response (or binding) is reduced by half. AUC indicates the area under the dose-response curve, and Amax indicates the maximal effect level. We also provide the raw data before extracting these measures.

File Name: Supplementary Data 2
Description: **The 160 drugs in our customized drug panel and their functional classes.**

File Name: Supplementary Data 3
Description: **Clinical information of the 30 AML patient samples.**

File Name: Supplementary Data 4
Description: **MERGE score, five driver features, and the significant p-value count for each gene.**

File Name: Supplementary Data 5
Description: **Processed RNA-seq data and drug sensitivity AUC measures from the 12 refractory AML patient samples used for validation.**

File Name: Supplementary Data 6
Description: **For each drug class shown in the columns of Fig. 6a, the list of significant genes in the order of the MERGE score, and the corresponding gene properties identified by the MERGE algorithm.** Class names are shown in red. We include only the genes with at least one consistent p-value in the cell lines and that are specific the drug class. The columns represent the following: gene name; gene's MERGE score; p-value for the significance of association of the gene with each of the drugs in the drug class; the number of significantly associated drugs; the number of significantly associated drugs that are consistent in the cell lines; Fisher's exact test p-value for the drug class specificity; and the gene's five individual driver feature scores.
